# Determination of critical community size from an HIV/AIDS model

**DOI:** 10.1371/journal.pone.0244543

**Published:** 2021-01-28

**Authors:** Sarmistha Das, Pramit Ghosh, Sandip Banerjee, Saumyadipta Pyne, Joydev Chattopadhyay, Indranil Mukhopadhyay

**Affiliations:** 1 Human Genetics Unit, Indian Statistical Institute, Kolkata, West Bengal, India; 2 Deben Mahata Government Medical College & Hospital, Purulia, West Bengal, India; 3 Department of Mathematics, Indian Institute of Technology, Roorkee, Uttarakhand, India; 4 Public Health Dynamics Lab and Department of Biostatistics, Graduate School of Public Health, University of Pittsburgh, Pittsburgh, Pennsylvania, United States of America; 5 Health Analytics Network, Pittsburgh, Pennsylvania, United States of America; 6 Agricultural and Ecological Research Unit, Indian Statistical Institute, Kolkata, West Bengal, India; Montclair State University, UNITED STATES

## Abstract

After an epidemic outbreak, the infection persists in a community long enough to engulf the entire susceptible population. Local extinction of the disease could be possible if the susceptible population gets depleted. In large communities, the tendency of eventual damp down of recurrent epidemics is balanced by random variability. But, in small communities, the infection would die out when the number of susceptible falls below a certain threshold. Critical community size (CCS) is considered to be the mentioned threshold, at which the infection is as likely as not to die out after a major epidemic for small communities unless reintroduced from outside. The determination of CCS could aid in devising systematic control strategies to eradicate the infectious disease from small communities. In this article, we have come up with a simplified computation based approach to deduce the CCS of HIV disease dynamics. We consider a deterministic HIV model proposed by Silva and Torres, and following Nåsell, introduce stochasticity in the model through time-varying population sizes of different compartments. Besides, Metcalf’s group observed that the relative risk of extinction of some infections on islands is almost double that in the mainlands i.e. infections cease to exist at a significantly higher rate in islands compared to the mainlands. They attributed this phenomenon to the greater recolonization in the mainlands. Interestingly, the application of our method on demographic facts and figures of countries in the AIDS belt of Africa led us to expect that existing control measures and isolated locations would assist in temporary eradication of HIV infection much faster. For example, our method suggests that through systematic control strategies, after 7.36 years HIV epidemics will temporarily be eradicated from different communes of island nation Madagascar, where the population size falls below its CCS value, unless the disease is reintroduced from outside.

## Introduction

Periodicity in the recurrence of local epidemics has attracted mathematicians and epidemiologists for centuries. Soper [1] pointed out that the time to extinction of infection and the size of the susceptible population are possibly the responsible candidates behind the periodicity. The persistence of a disease in a community, often measured by the time for an epidemic to die out, is a major concern to date. After an epidemic outbreak, the infection persists in the community long enough to engulf the entire susceptible population. Local extinction of the disease could be possible if the susceptible population gets depleted. The success of Hamer-Soper model [1] in predicting periodicity of epidemics from simple assumptions, led Bartlett [2–4] to observe a threshold for the susceptible population size. If the susceptible population is equal to that threshold, there is a 50% chance that the infection would cease to exist after the corresponding mean fade-out time unless the disease is reintroduced from outside. He analyzed this threshold using a semi-stochastic version of the Hamer-Soper model and established time to extinction of the epidemic using quasi stationarity. Bartlett’s model sufficiently explained the undamped epidemic outbreaks and its extinction. Bartlett applied the semi-stochastic model for the recurrent measles outbreak in England and Wales. He observed that in large communities, the tendency of eventual damp down of recurrent epidemics is balanced by random variability; but, in small communities, the infection would die out when the size of the susceptible population falls below a certain threshold. These led Bartlett to define Critical Community Size (CCS) as a threshold size of the susceptible population in a small community, at which infection is as likely as not to die out, after a major epidemic, unless reintroduced from outside. The idea is that, if the size of susceptible population drops below that threshold, the pathogen might cease to exist in the population.

Apart from the works by Andersson and Britton [5], scarcely significant mathematical contributions were made in this direction until Nåsell [6] reformulated CCS for Bartlett’s Susceptible-Infected (SI) model using quasi stationarity under more realistic assumptions. Nåsell [6, 7] used a fully stochastic model to study the time to extinction of an epidemic for a number of disease models. The model and method proposed by Nåsell [6] is based on a standard SI model that involves a system of two differential equations. This model can easily be extended to an SIR model where the population size remains constant at any time, as this SIR model can be reduced to a two-equation model and the above method is applicable without any significant modification. Nåsell [6] also focused to unleash the potential of CCS as a control strategy for the epidemic outbreak. Later, Metcalf et al. [8] mentioned this potential of CCS. But, calculation of CCS is limited mostly to models based on a two-equation system of differential equations or measles type diseases. One predominant reason is that the analytical formulation of CCS for complex infectious diseases is quite cumbersome. The absence of any method to calculate CCS for higher-order models motivated us to develop a cost (in terms of time) effective technique to evaluate CCS. In this paper, we propose a simplified computation based approach for rapid calculation of CCS, that could emphasize prompt formulation of control strategies and/or policies at the time of disease outbreak.

Technically, this paper determines the time to extinction of an epidemic outbreak theoretically and also develops a computation based approximate formulation of CCS using the diffusion approximation. The application of our method is free from any restriction on the number of variables in the system that defines the disease dynamics. To demonstrate our method we consider an HIV model by Silva, Torres, and Djordjevic [9–11] that describes the disease dynamics using a system of four differential equations. Since infectious diseases mostly have a stochastic nature [12–14], we consider a stochastic version of the HIV disease dynamics. Following Nåsell, we introduce stochasticity through time-varying population sizes of different compartments in the deterministic HIV model [9]. Next, we calculate the mean time to extinction of the HIV epidemic and compute approximate value of CCS using diffusion approximation based on the Ornstein-Uhlenbeck process.

In this paper, we have cited the HIV epidemic case of Madagascar, in Sub-Saharan Africa because of a special reason. In spite of less remarkable progress in the 90-90-90 HIV treatment target of the Joint United Nations Programme on HIV and AIDS (UNAIDS), Madagascar has shown a much lower prevalence and incidence rate of HIV infection, compared to some other African countries. It is interesting to note that, despite being in the part of Africa where HIV is in a state of epidemic, it has much lower rates of prevalence and incidence. Incidentally, Madagascar is situated in a geographically isolated location, separated by 60 km of water from the other eastern and southern countries that lie in the mainland of the African continent. This observation echoes a fact from Metcalf et al. [8], that says the relative risk of extinction of some infectious diseases on islands is almost double that of those in the mainland. They also suggest that persistence of the infection within a region in the mainland is fuelled constantly by recolonization and infections cease to exist at significantly higher rates in islands where access to and from the mainland is much less. This fascinating phenomenon led us to postulate that, in conjunction with UNAIDS controlling strategies if it is possible to keep the HIV susceptible population of Madagascar small or rather below CCS, effective and rapid eradication of HIV might be possible there, after the mean extinction time of the disease. This is primarily based on the fact that the isolated geographical location of Madagascar might lead to lesser chances of reintroduction of the infection.

We, therefore, applied our method on the demographic facts and figures of Madagascar to obtain the CCS value and mean time to extinction of the disease. Our calculations suggest that, unless the disease is reintroduced from outside, after 7.36 years HIV epidemics might fade out from different cities or communes of the island nation where the susceptible population size drops below 4585, the CCS value of Madagascar. This time period of 7.36 years could appear astonishing as HIV has become a long-standing epidemic situation in the countries of the Sub-Saharan AIDS belt. But there is a strong possibility that the idea of CCS would propel the controlling strategies of UNAIDS to eradicate the disease in the near future. This work suggests that the systematic spreading of control measures could lead to accelerated progress to achieve the 90-90-90 target of UNAIDS for HIV eradication. Such progress shall prevent the HIV epidemic in Madagascar unless the infection is reintroduced from outside. Moreover, countries like Uganda, Malawi (located in Eastern and Southern Africa) are separated from their neighboring countries by water bodies such as fresh water lakes. These countries have lower rates of prevalence and incidence compared to landlocked countries such as Eswatini (in Southern Africa). Notably, these rates are still much higher than that of Madagascar.

To date, CCS has largely been a part of mathematical theory only. But implementing CCS as controlling strategies of epidemics might prove beneficial in the future. Therefore, to utilize the potential that CCS holds, it is important to determine its value promptly for any disease dynamics. This motivated us to calculate CCS (approximately) using simplified computations. Our method will help in finding country-specific CCS and time to extinction of the disease, using real demographic facts and figures for HIV infection in different countries as parameter values. We emphasize the potential utility of CCS to guide public health practitioners to develop appropriate area-specific control strategies and interventions especially when they are limited, before or after an epidemic outbreak.

## Materials and methods

### Model formulation

We work with the model proposed by Silva and Torres [9] that explains the dynamics of HIV infection assuming homogenous mixing within communities. Here the entire population is divided into four compartments. These compartments are mutually exclusive in the sense that no person can belong to more than one compartment at any time point. The four compartments are: susceptible individuals (*S*), individuals infected with HIV but having no clinical symptoms of AIDS (*I*), individuals infected with AIDS and having ART treatment as they are supposed to belong to chronic stage (*C*), and individuals infected with HIV having clinical symptoms of AIDS (*A*). Note that a person belonging to *I* may transmit HIV to other individuals although he/she has no symptoms of AIDS. Under this situation, the model we consider is,

$$\frac{dS}{dt}=\Lambda -\beta (I\left(t\right)+\eta_{C}C\left(t\right)+\eta_{A}A\left(t\right))\frac{S(t)}{N}-\mu S\left(t\right)$$ (1)

$$\frac{dI}{dt}=\beta (I\left(t\right)+\eta_{C}C\left(t\right)+\eta_{A}A\left(t\right))\frac{S(t)}{N}-\left(\rho +\phi +\mu \right)I\left(t\right)+\omega C\left(t\right)+\alpha A\left(t\right)$$ (2)

$$\frac{dC}{dt}=\phi I(t)-(\omega +\mu )C(t)$$ (3)

$$\frac{dA}{dt}=\rho I(t)-(\alpha +\mu +d)A(t)$$ (4)

Here *β* represents the contact rate for HIV transmission, *η*_*A*_ (≥ 1) is the relative infectiousness of individuals with AIDS symptoms compared to those infected with HIV but no AIDS symptoms, and *η*_*C*_ (≤ 1) is the rate of partial restoration of immune function of HIV infected individuals who are correctly treated under ART. *ρ* and *ϕ* are the rates at which individuals in class *I* move to *A* and *C* respectively. *α* and *ω* are the respective rates at which individuals from class *A* and class *C* move to *I*. We assume that *d* is the HIV induced death rate while *μ* is the natural death rate in the population.

We consider the total population as *N*(*t*) = *S*(*t*) + *I*(*t*) + *A*(*t*) + *C*(*t*) implying that $\frac{dN(t)}{dt}=\Lambda -\mu N\left(t\right)-dA\left(t\right)$. It can be easily shown that $N\left(t\right)\leq \frac{\Lambda }{\mu }$.

### Basic reproduction number

We use the approach of next generation matrix [15] to calculate *R*_0_. Note that the rate of new infections coming in the compartments *I*, *C* and *A* are $\beta \left(I+\eta_{C}C+\eta_{A}A\right)\frac{S}{N}$, 0 and 0 respectively. On the other hand, new infections moving out of the compartments *I*, *C*, and *A* are (*ρ* + *ϕ* + *μ*)*I* − *ωC* − *αA*, (*ω* + *μ*)*C* − *ϕI* and (*α* + *μ* + *d*)*A* − *ρI* respectively. Thus following [9, 15], we have basic reproduction number (*R*_0_) as:

$$\begin{array}{c} R_{0}=\frac{S^{0}\beta }{N}\frac{\xi_{2}\left(\xi_{1}+\rho \eta_{A}\right)+\eta_{C}\phi \xi_{1}}{\mu (\xi_{2}+\left(\rho +\xi_{1}\right)+\phi \xi_{1}+\rho d)+\rho \omega d}=\frac{S^{0}}{N}\frac{\zeta }{\gamma }\approx \frac{\zeta }{\gamma } \end{array}$$ (5)

where *ζ* = *β*(*ξ*_2_(*ξ*_1_ + *ρη*_*A*_) + *η*_*C*_ *ϕξ*_1_), *γ* = *μ*(*ξ*_2_ + (*ρ* + *ξ*_1_) + *ϕξ*_1_ + *ρd*) + *ρωd*, *ξ*_1_ = *α* + *μ* + *d* and *ξ*_2_ = *ω* + *μ*.

### Stochastic model and quasi-stationarity

Evaluation of CCS depends on the time to extinction of disease based on the given model. In order to find the time to extinction, we now construct the fully stochastic version of the model (1)—(4). Next, we write the Kolmogorov forward equation for this stochastic model. This would be the key step for further downstream analysis.

First, we note the nature of transitions and the respective transition rates from one compartment to another (Table 1).

**Table 1:** Chart for transition rates.

Event	Transition	Transition rate
Immigration of susceptibles (S)	(*s*, *i*, *c*, *a*) → (*s* + 1, *i*, *c*, *a*)	λ_1_(*s*, *i*, *c*, *a*) = *μN*
Death of susceptibles (S)	(*s*, *i*, *c*, *a*) → (*s* − 1, *i*, *c*, *a*)	λ_2_(*s*, *i*, *c*, *a*) = *μs*
Susceptible (S) to Infected (I)	(*s*, *i*, *c*, *a*) → (*s* − 1, *i* + 1, *c*, *a*)	λ_3_(*s*, *i*, *c*, *a*) = *βsi*/*N*
Susceptible (S) to Infected under treatment (C)	(*s*, *i*, *c*, *a*) → (*s* − 1, *i*, *c* + 1, *a*)	λ_4_(*s*, *i*, *c*, *a*) = *βη*_*C*_ *sc*/*N*
Susceptible (S) to Infected with AIDS (A)	(*s*, *i*, *c*, *a*) → (*s* − 1, *i*, *c*, *a* + 1)	λ_5_(*s*, *i*, *c*, *a*) = *βη*_*A*_ *sa*/*N*
Infected (I) to Infected under treatment(C)	(*s*, *i*, *c*, *a*) → (*s*, *i* − 1, *c* + 1, *a*)	λ_6_(*s*, *i*, *c*, *a*) = *ϕi*
Infected (I) to Infected with AIDS (A)	(*s*, *i*, *c*, *a*) → (*s*, *i* − 1, *c*, *a* + 1)	λ_7_(*s*, *i*, *c*, *a*) = *ρi*
Death of Infected (I)	(*s*, *i*, *c*, *a*) → (*s*, *i* − 1, *c*, *a*)	λ_8_(*s*, *i*, *c*, *a*) = *μi*
Infected under treatment (C) to infected (I)	(*s*, *i*, *c*, *a*) → (*s*, *i* + 1, *c* − 1, *a*)	λ_9_(*s*, *i*, *c*, *a*) = *ωc*
Death of Infected under treatment(C)	(*s*, *i*, *c*, *a*) → (*s*, *i*, *c* − 1, *a*)	λ_10_(*s*, *i*, *c*, *a*) = *μc*
Infected with AIDS symptoms (A) to Infected (I)	(*s*, *i*, *c*, *a*) → (*s*, *i* + 1, *c*, *a* − 1)	λ_11_(*s*, *i*, *c*, *a*) = *αa*
Death of Infected with AIDS symptoms (A)	(*s*, *i*, *c*, *a*) → (*s*, *i*, *c*, *a* − 1)	λ_12_(*s*, *i*, *c*, *a*) = (*μ* + *d*)*a*

Here, *s* = *S*/*N*, *i* = *I*/*N*, *c* = *C*/*N*, *a* = *A*/*N*. Then the Kolmogorov forward equation becomes,

$$\begin{array}{cc} p_{s,i,c,a}^{\prime }\left(t\right) & =\lambda_{1}\left(s-1,i,c,a\right)p_{s-1,i,c,a}+\lambda_{2}\left(s+1,i,c,a\right)p_{s+1,i,c,a}\\ & +\lambda_{3}\left(s+1,i-1,c,a\right)p_{s+1,i-1,c,a}+\lambda_{4}\left(s+1,i,c-1,a\right)p_{s+1,i,c-1,a}\\ & +\lambda_{5}\left(s+1,i,c,a-1\right)p_{s+1,i,c,a-1}+\lambda_{6}\left(s,i+1,c-1,a\right)p_{s,i+1,c-1,a}\\ & +\lambda_{7}\left(s,i+1,c,a-1\right)p_{s,i+1,c,a-1}+\lambda_{8}\left(s,i+1,c,a\right)p_{s,i+1,c,a}\\ & +\lambda_{9}\left(s,i-1,c+1,a\right)p_{s,i-1,c+1,a}+\lambda_{10}\left(s,i,c+1,a\right)p_{s,i,c+1,a}\\ & +\lambda_{11}\left(s,i-1,c,a+1\right)p_{s,i-1,c,a+1}+\lambda_{12}\left(s,i,c,a+1\right)p_{s,i,c,a+1}-\kappa \left(s,i,c,a\right)p_{s,i,c,a} \end{array}$$ (6)

where $\kappa \left(s,i,c,a\right)=\sum_{i=1}^{12}\lambda_{i}\left(s,i,c,a\right)$ and *p*_*s*,*i*,*c*,*a*_(*t*) = *P*[*S*(*t*) = *s*, *I*(*t*) = *i*, *C*(*t*) = *c*, *A*(*t*) = *a*].

Now, we find quasi-stationarity by conditioning on non-extinction of the disease. So we write,

$$\begin{array}{c} q_{s,i,c,a}\left(t\right)=P[S(t)=s,I(t)=i,C(t)=c,A(t)=a|(I(t),C(t),A(t))\neq (0,0,0)]=\frac{p_{s,i,c,a}\left(t\right)}{1-p_{•000}\left(t\right)} \end{array}$$

where $p_{•000}\left(t\right)=\sum_{s=0}^{\infty }P[S(t)=s,I(t)=0,C(t)=0,A(t)=0]=\sum_{s=0}^{\infty }p_{s,0,0,0}\left(t\right)$.

Differentiating *q*_*s*,*i*,*c*,*a*_(*t*) with respect to *t*, we have,

$$\begin{array}{c} q_{s,i,c,a}^{\prime }\left(t\right)=\frac{p_{s,i,c,a}^{\prime }\left(t\right)}{1-p_{•000}\left(t\right)}+\frac{p_{s,i,c,a}\left(t\right)}{{\left(1-p_{•000}\left(t\right)\right)}^{2}}.p_{•000}^{\prime }\left(t\right). \end{array}$$ (7)

Now, from Eq (6), we have, after simplification,

$$\begin{array}{cc} p_{•000}^{\prime }\left(t\right) & =\sum_{s=0}^{\infty }[\mu Np_{s-1,0,0,0}\left(t\right)+\mu \left(s+1\right)p_{s+1,0,0,0}\left(t\right)+\mu \left(0+1\right)p_{s,1,0,0}\left(t\right)\\ & +\mu \left(0+1\right)p_{s,0,1,0}\left(t\right)+\left(\mu +d\right)\left(0+1\right)p_{s,0,0,1}\left(t\right)]-\left(\mu N+\mu s\right)\sum_{s=0}^{\infty }p_{s,0,0,0}\left(t\right)\\ & =\mu p_{•1\;0\;0}\left(t\right)+\mu p_{•0\;1\;0}\left(t\right)+\left(d+\mu \right)p_{•0\;0\;1}\left(t\right)=p_{•}^{\left(d,\mu \right)}\left(t\right)\;\;\;\left(say\right). \end{array}$$ (8)

From Eqs (7) and (8) we have,

$$\begin{array}{cc} q_{s,i,c,a}^{\prime }\left(t\right) & =\frac{p_{s,i,c,a}^{\prime }\left(t\right)}{1-p_{•0\;0\;0}\left(t\right)}+\frac{p_{s,i,c,a}\left(t\right)}{{\left(1-p_{•0\;0\;0}\left(t\right)\right)}^{2}}.p_{•0\;0\;0}^{\prime }\left(t\right)\\ & =\frac{p_{s,i,c,a}^{\prime }\left(t\right)}{1-p_{•0\;0\;0}\left(t\right)}+\frac{p_{s,i,c,a}\left(t\right)}{1-p_{•0\;0\;0}\left(t\right)}.\frac{p_{•}^{\left(d,\mu \right)}\left(t\right)}{1-p_{•0\;0\;0}\left(t\right)}\\ & =\frac{p_{s,i,c,a}^{\prime }\left(t\right)}{1-p_{•0\;0\;0}\left(t\right)}+\frac{p_{s,i,c,a}\left(t\right)}{1-p_{•0\;0\;0}\left(t\right)}.q_{•}^{\left(d,\mu \right)}\left(t\right),\;\;\;\left(say\right) \end{array}$$ (9)

$$\begin{array}{c} \text{where}\;q_{•}^{\left(d,\mu \right)}\left(t\right)=\frac{p_{•}^{\left(d,\mu \right)}\left(t\right)}{1-p_{•0\;0\;0}\left(t\right)} \end{array}$$ (10)

Now, following Nåsell [6, 7] we have,

$$\begin{array}{cc} & q_{s,i,c,a}^{\prime }\left(t\right)=0\\ & \Rightarrow p_{s,i,c,a}^{\prime }\left(t\right)=-\frac{p_{s,i,c,a}\left(t\right)}{\left(1-p_{•0\;0\;0}\left(t\right)\right)}.q_{•}^{\left(d,\mu \right)}\left(t\right)\left(1-p_{•0\;0\;0}\left(t\right)\right)=-q_{•}^{\left(d,\mu \right)}\left(t\right)p_{s,i,c,a}\left(t\right)\\ & ∴p_{s,i,c,a}\left(t\right)=ce^{-q_{•}^{\left(d,\mu \right)}.t}=q_{•}^{\left(d,\mu \right)}e^{-q_{•}^{\left(d,\mu \right)}.t}\;\; \end{array}$$ (11)

Let *τ*_*Q*_ be the time to extinction when the initial distribution is equal to the quasi-stationary distribution. Therefore, we have,

$$\begin{array}{c} E\left(\tau_{Q}\right)=\frac{1}{q_{•}^{\left(d,\mu \right)}}. \end{array}$$ (12)

### Equilibrium points

The disease-free equilibrium is obtained as: $\Sigma_{0}=\left(S^{0},I^{0},C^{0},A^{0}\right)=(\frac{\Lambda }{\mu },0,0,0)$. To find the other endemic equilibrium, if exists, we put *N* = *N*(0), *x*_1_(*t*) = *S*(*t*)/*N*, *x*_2_(*t*) = *I*(*t*)/*N*, *x*_3_(*t*) = *C*(*t*)/*N*, and *x*_4_(*t*) = *A*(*t*)/*N*. The equilibrium point is obtained by equating the first differentiation in Eqs (1)–(4) to zero [9, 10], i.e.

$$\begin{array}{ccc} & x_{1}^{\prime }\left(t\right)=\mu -\beta (x_{2}\left(t\right)+\eta_{C}x_{3}\left(t\right)+\eta_{A}x_{4}\left(t\right))x_{1}-\mu x_{1}\left(t\right)=0 & \end{array}$$ (13)

$$\begin{array}{ccc} & x_{2}^{\prime }\left(t\right)=\beta (x_{2}\left(t\right)+\eta_{C}x_{3}\left(t\right)+\eta_{A}x_{4}\left(t\right))x_{1}-\left(\rho +\phi +\mu \right)x_{2}\left(t\right)+\omega x_{3}\left(t\right)+\alpha x_{4}\left(t\right)=0 & \end{array}$$ (14)

$$\begin{array}{ccc} & x_{3}^{\prime }\left(t\right)=\phi x_{2}\left(t\right)-\left(\omega +\mu \right)x_{3}\left(t\right)=0 & \end{array}$$ (15)

$$\begin{array}{cc} & x_{4}^{\prime }\left(t\right)=\rho x_{2}\left(t\right)-\left(\alpha +\mu +d\right)x_{4}\left(t\right)=0 \end{array}$$ (16)

For simplicity we use the notations: *x*_*j*_(*t*) = *x*_*j*_ for *j* = 1, …, 4, *ξ*_1_ = *α* + *μ* + *d*, *ξ*_2_ = *ω* + *μ*, *γ* = *ξ*_1_ *ξ*_2_(*ρ* + *ϕ* + *μ*) − *ωϕξ*_1_ − *ραξ*_2_, *ζ* = *β*(*ξ*_1_ *ξ*_2_+ *η*_*C*_ *ϕξ*_1_+ *η*_*A*_ *ρξ*_2_).

Then solving Eqs (13)–(16), we have the endemic equilibrium as:

$${\hat{x}}_{1}=\frac{\gamma }{\zeta }=\frac{1}{R_{0}}$$ (17)

$${\hat{x}}_{2}=\frac{\left(\zeta -\gamma \right)}{\zeta \gamma }.\mu \xi_{1}\xi_{2}=\left(R_{0}-1\right)\frac{\mu }{\zeta }.\xi_{1}\xi_{2}$$ (18)

$${\hat{x}}_{3}=\frac{\left(\zeta -\gamma \right)}{\zeta \gamma }.\mu \phi \xi_{1}=\left(R_{0}-1\right)\frac{\mu }{\zeta }.\phi \xi_{1}$$ (19)

$${\hat{x}}_{4}=\frac{\left(\zeta -\gamma \right)}{\zeta \gamma }.\mu \rho \xi_{2}=\left(R_{0}-1\right)\frac{\mu }{\zeta }.\rho \xi_{2}$$ (20)

### Diffusion approximation

We consider a diffusion approximation to the stochastic version of our model (1)—(4). This would allow us to approximately evaluate the quasi-stationary distribution using a multivariate normal distribution when *N* is large and *R*_0_ is greater than 1. First we find the multivariate normal distribution corresponding to the state variables. Let the changes in the scaled state variables *x*_1_, *x*_2_, *x*_3_, and *x*_4_ during the time interval (*t*, *t* + *δt*) be denoted by *δx*_1_, *δx*_2_, *δx*_3_, and *δx*_4_ respectively, where *δx*_*i*_(*t*) = *x*_*i*_(*t* + *δt*) − *x*_*i*_(*t*), 1, 2, 3, 4.

Under the assumptions of the original process on the sequence of transitions, we evaluate the mean vector and covariance matrix for *δx*_*i*_ (*i* = 1, 2, 3, 4) during the time interval (*t*, *t* + *δt*) as follows. At first, we assume that we are in the state (*S*, *I*, *C*, *A*). So, the possible transitions from this state are:

(a) *S* increases by 1 at the rate *μ*
(b) *S* decreases by 1 at the rate *μ*
(c) *S* decreases by 1 and *I* increases by 1 at the rate *βSI*/*N*
(d) *S* decreases by 1 and *I* increases by 1 at the rate *βη*_*C*_ *SC*/*N*
(e) *S* decreases by 1 and *I* increases by 1 at the rate *βη*_*A*_ *SA*/*N*
(f) *I* decreases by 1 and *C* increases by 1 at the rate *ϕI*
(g) *I* decreases by 1 and *A* increases by 1 at the rate *ρI*
(h) *I* decreases by 1 at the rate *μI*
(i) *C* decreases by 1 and *I* increases by 1 at the rate *ωC*
(j) *C* decreases by 1 at the rate *μC*
(k) *A* decreases by 1 and *I* increases by 1 at the rate *αA*
(l) *A* decrease by 1 at the rate (*μ* + *d*)*A*.

The random variable *δx*_1_ equals $\frac{1}{N}$ in cases (*a*), $-\frac{1}{N}$ in cases (*b*), (*c*), (*d*), (*e*), and 0 in other cases. Similarly, *δx*_2_ equals $\frac{1}{N}$ in cases (*c*), (*d*), (*e*), (*i*), (*k*), $-\frac{1}{N}$ in cases (*f*), (*g*), (*h*), and 0 in other cases. *δx*_3_ equals $\frac{1}{N}$ in cases (*f*), $-\frac{1}{N}$ in cases (*i*), (*j*), and 0 in other cases. *δx*_4_ equals $\frac{1}{N}$ in cases (*g*), $-\frac{1}{N}$ in cases (*k*), (*l*), and 0 in other cases. Denoting ***x*** = (*x*_1_, *x*_2_, *x*_3_, *x*_4_)′ we have,

$$\begin{array}{cc} & E(\delta x)=b(x)\delta t+o(\delta t) \end{array}$$

$$\begin{array}{c} \text{where}\;b\left(x\right)=(\begin{array}{c} \mu -\beta \left(x_{2}+\eta_{C}x_{3}+\eta_{A}x_{4}\right)x_{1}-\mu x_{1}\\ \beta \left(x_{2}+\eta_{C}x_{3}+\eta_{A}x_{4}\right)x_{1}-\left(\rho +\phi +\mu \right)x_{2}+\omega x_{3}+\alpha x_{4}\\ \phi x_{2}-\left(\omega +\mu \right)x_{3}\\ \rho x_{2}-\left(\alpha +\mu +d\right)x_{4} \end{array}) \end{array}$$ (21)

Now to derive the covariance matrix we need to find the Jacobian matrix at point ***x***, which is obtained from *b*(***x***) as,

$$\begin{array}{c} B\left(x\right)=\frac{\partial b(x)}{\partial x}=(\begin{array}{cccc} -\beta (x_{2}+\eta_{C}x_{3}+\eta_{A}x_{4})-\mu & -\beta x_{1} & -\beta \eta_{C}x_{1} & -\beta \eta_{A}x_{1}\\ \beta (x_{2}+\eta_{C}x_{3}+\eta_{A}x_{4}) & \beta x_{1}-\left(\rho +\phi +\mu \right) & \beta \eta_{C}x_{1}+\omega & \beta \eta_{A}x_{1}+\alpha \\ 0 & \phi & -(\omega +\mu ) & 0\\ 0 & \rho & 0 & -(\alpha +\mu +d) \end{array}) \end{array}$$

Then we approximate *B*(***x***) at equilibrium point $\hat{x}={\left({\hat{x}}_{1},{\hat{x}}_{2},{\hat{x}}_{3},{\hat{x}}_{4}\right)}^{\prime }$ by $B(\hat{x})$. So, covariance matrix of *δx* = (*δx*_1_, *δ* ***x***_2_, *δx*_3_, *δx*_4_)′ is,

$V\left(\delta x\right)=\frac{1}{N}S\left(x\right)\delta t+o\left(\delta t\right)$ where,

$$\begin{array}{c} S\left(x\right)=(\begin{array}{cccc} \beta \left(x_{2}+\eta_{C}x_{3}+\eta_{A}x_{4}\right)x_{1} & -\beta x_{1}x_{2} & -\beta \eta_{C}x_{1}x_{3} & -\beta \eta_{A}x_{1}x_{4}\\ \mu^{\prime }+\mu x_{1} & & & \\ -\beta x_{1}x_{2} & \beta \left(x_{2}+\eta_{C}x_{3}+\eta_{A}x_{4}\right)x_{1} & -\phi x_{2}-\omega x_{3} & -\rho x_{2}-\alpha x_{4}\\ & +\left(\rho +\phi +\mu \right)x_{2}+\omega x_{3}+\alpha x_{4} & & \\ -\beta \eta_{C}x_{1}x_{3} & -\phi x_{2}-\omega x_{3} & \phi x_{2}+\left(\omega +\mu \right)x_{3} & 0\\ -\beta \eta_{A}x_{1}x_{4} & -\rho x_{2}-\alpha x_{4} & 0 & \rho x_{2}\\ & & & +\left(\alpha +\mu +d\right)x_{4} \end{array}) \end{array}$$

We again approximate *S*(***x***) by $S(\hat{x})$, where $\hat{x}$ is the equilibrium point. For large *N*, the process $\sqrt{N}\left(x\left(t\right)-\hat{x}\right)$ is approximated by a multivariate Ornstein-Uhlenbeck (O-U) process [16], with a local drift matrix $B(\hat{x})$ and local covariance matrix $S(\hat{x})$.

The stationary distribution of this O-U process approximates the quasi-stationary distribution. It is approximately normal with mean zero and covariance matrix Σ, where Σ is obtained by solving

$$\begin{array}{c} B\left(\hat{x}\right)\Sigma +\Sigma B^{\prime }\left(\hat{x}\right)=-S\left(\hat{x}\right). \end{array}$$ (22)

Exact analytical solution for Σ is not straightforward [5]. However, since we are interested in calculating the CCS, we can easily solve the Eq (22) numerically using the parameter values and the equilibrium point.

Let *σ*_*ij*_ be the solution of the (*i*, *j*)th element of Σ, where *i*, *j* = 1, …, 4. The diffusion approximation guides us to consider appropriate mean and variance-covariance matrix for the joint distribution of *x*(*t*). Thus we have,

$$\begin{array}{c} \sqrt{N}\left(x\left(t\right)-\hat{x}\right)∼N_{4}\left(0,\Sigma \right),\;\text{with}\;x\left(t\right)={\left(x_{1}\left(t\right),x_{2}\left(t\right),x_{3}\left(t\right),x_{4}\left(t\right)\right)}^{\prime },\hat{x}={\left({\hat{x}}_{1},{\hat{x}}_{2},{\hat{x}}_{3},{\hat{x}}_{4}\right)}^{\prime }, \end{array}$$

$$\begin{array}{c} \text{and}\;\Sigma =(\begin{array}{cccc} \sigma_{11} & \sigma_{12} & \sigma_{13} & \sigma_{14}\\ \sigma_{21} & \sigma_{22} & \sigma_{23} & \sigma_{24}\\ \sigma_{31} & \sigma_{32} & \sigma_{33} & \sigma_{34}\\ \sigma_{41} & \sigma_{42} & \sigma_{43} & \sigma_{44} \end{array}) \end{array}$$ (23)

The approximation for quasi-stationary distribution is obtained by using conditional truncated distributions of the above multivariate normal distribution. Thus in order to evaluate *p*_•000_, *p*_•100_, *p*_•010_, and *p*_•001_ we need to use a result from conditional truncated multivariate normal distribution, which is given below as theorem 1 (for proof see S1 Appendix). Even then, it is extremely difficult, if not impossible [5], to get an exact expression for *q*_•_(*d*, *μ*). So, we evaluate an approximate expression of *q*_•_(*d*, *μ*) using Result 1 (for proof see S1 Appendix).

**Theorem 1** *Let* ***Y*** ∼ *N*_*p*_(***μ***, Σ) *and write* $y={\left(y_{1}^{\prime },y_{2}^{\prime }\right)}^{\prime }$, $\mu ={\left(\mu_{1}^{\prime },\mu_{2}^{\prime }\right)}^{\prime }$, *and* $\Sigma =(\begin{array}{cc} \Sigma_{11} & \Sigma_{12}\\ \Sigma_{21} & \Sigma_{22} \end{array})$. *Suppose instead of* $R^{p}$, ***Y*** *is defined only on a truncated support* ***c*** < ***y*** < ***d***. *Consider the partitions* $c={\left(c_{1}^{\prime },c_{2}^{\prime }\right)}^{\prime }$ *and* $d={\left(d_{1}^{\prime },d_{2}^{\prime }\right)}^{\prime }$. *Then, the conditional distribution of* ***Y***_1_ *given* ***y***_2_ *is given by*

$$\begin{array}{c} f^{*}\left(y_{1}|y_{2}\right)=\frac{f(y_{1}|y_{2})}{\int_{c_{1}}^{d_{1}}f\left(y_{1}|y_{2}\right)dy_{1}} \end{array}$$ (24)

*where f*(***y***_1_|***y***_2_) *is the conditional probability density function of* ***Y***_1_ *given* ***y***_2_ *i.e*. $Y_{1}|y_{2}∼N_{q}\left(\mu_{1}+\Sigma_{12}\Sigma_{22}^{-1}\left(y_{2}-\mu_{2}\right),\Sigma_{11.2}=\Sigma_{11}-\Sigma_{12}\Sigma_{22}^{-1}\Sigma_{21}\right)$

**Result 1** *Let* $\phi \left(x\right)=\frac{1}{\sqrt{2\pi }}e^{-x^{2}/2}$ *and* $\Phi \left(x\right)=\int_{-\infty }^{x}\phi \left(t\right)dt\;for\;any\;x\in \left(-\infty ,\infty \right)$. *Then, an approximate expression of* $q_{•}^{\left(d,\mu \right)}$ *is given as*,

$$\begin{array}{c} q_{•}^{\left(d,\mu \right)}=\frac{\mu p_{•100}+\mu p_{•010}+\left(d+\mu \right)p_{•001}}{1-p_{•000}} \end{array}$$ (25)

$$\begin{array}{cc} where & p_{•100}\approx \frac{1}{2N\sqrt{\sigma_{22}^{*}}}\frac{\phi (\frac{{\hat{x}}_{2}^{*}}{\sqrt{\sigma_{22}^{*}}})}{\Phi (\frac{{\hat{x}}_{2}^{*}}{\sqrt{\sigma_{22}^{*}}})}.\frac{1}{2N\sqrt{\sigma_{33}^{*}}}\frac{\phi (\frac{{\hat{x}}_{3}^{*}}{\sqrt{\sigma_{33}^{*}}})}{\Phi (\frac{{\hat{x}}_{3}^{*}}{\sqrt{\sigma_{33}^{*}}})}.\frac{1}{2N\sqrt{\sigma_{44}}}\frac{\phi (\frac{{\hat{x}}_{4}}{\sqrt{\sigma_{44}}})}{\Phi (\frac{{\hat{x}}_{4}}{\sqrt{\sigma_{44}}})}\\ & p_{•010}\approx \frac{1}{2N\sqrt{\sigma_{33}^{**}}}\frac{\phi (\frac{{\hat{x}}_{3}^{**}}{\sqrt{\sigma_{3}^{**}}})}{\Phi (\frac{{\hat{x}}_{3}^{**}}{\sqrt{\sigma_{33}^{**}}})}.\frac{1}{2N\sqrt{\sigma_{22}^{**}}}\frac{\phi (\frac{{\hat{x}}_{2}^{**}}{\sqrt{\sigma_{2}^{**}}})}{\Phi (\frac{{\hat{x}}_{2}^{**}}{\sqrt{\sigma_{22}^{**}}})}.\frac{1}{2N\sqrt{\sigma_{44}}}\frac{\phi (\frac{{\hat{x}}_{4}}{\sqrt{\sigma_{44}}})}{\Phi (\frac{{\hat{x}}_{4}}{\sqrt{\sigma_{44}}})}\\ & p_{•001}\approx \frac{1}{2N\sqrt{\sigma_{44}^{***}}}\frac{\phi (\frac{{\hat{x}}_{4}^{***}}{\sqrt{\sigma_{44}^{***}}})}{\Phi (\frac{{\hat{x}}_{4}^{***}}{\sqrt{\sigma_{44}^{***}}})}.\frac{1}{2N\sqrt{\sigma_{22}^{***}}}\frac{\phi (\frac{{\hat{x}}_{2}^{***}}{\sqrt{\sigma_{22}^{***}}})}{\Phi (\frac{{\hat{x}}_{2}^{***}}{\sqrt{\sigma_{22}^{***}}})}.\frac{1}{2N\sqrt{\sigma_{33}}}\frac{\phi (\frac{{\hat{x}}_{3}}{\sqrt{\sigma_{33}}})}{\Phi (\frac{{\hat{x}}_{3}}{\sqrt{\sigma_{33}}})} \end{array}$$ (26)

*where* ${\hat{x}}_{i}^{*},\sigma_{ii}^{*}\;for\;i=2,3$, ${\hat{x}}_{i}^{**},\sigma_{ii}^{**}\;for\;i=2,3$, ${\hat{x}}_{i}^{***},\sigma_{ii}^{***}\;for\;i=2,4$ *are obtained from the truncated conditional distribution of multivariate normal distribution as given in* (23).

Once we find $q_{•}^{\left(d,\mu \right)}$, we have an expression for expected time to extinction $\hat{E}\left(\tau_{Q}\right)$ using Eq (12). However, this involves *N*, which is unknown. Now, our aim is to find the above expression in another way so that we can equate them and solve for *N*. So, following [6], if we can find the quasi-period (${\hat{T}}_{0}$), we may use the relation $\hat{E}\left(\tau_{Q}\right)\;\text{log}\;2={\hat{T}}_{0}$ to get the value of *N*, which is the critical community size.

We determine the quasi-period of the oscillation about the critical point using linearisation method [17]. Note that for our model, the linearised system about the equilibrium point $\hat{x}={\left({\hat{x}}_{1},{\hat{x}}_{2},{\hat{x}}_{3},{\hat{x}}_{4}\right)}^{\prime }$ can be written as:

$$\begin{array}{c} \frac{dx^{*}}{dt}=(\begin{array}{cccc} -\beta \left(1+\eta_{C}\frac{\phi }{\xi_{2}}+\eta_{A}\frac{\rho }{\xi_{1}}\right){\hat{x}}_{2}-\mu & -\frac{\beta }{R_{0}} & -\frac{\beta \eta_{C}}{R_{0}} & -\frac{\beta \eta_{A}}{R_{0}}\\ \beta \left(1+\eta_{C}\frac{\phi }{\xi_{2}}+\eta_{A}\frac{\rho }{\xi_{1}}\right){\hat{x}}_{2} & \frac{\beta }{R_{0}}-\left(\rho +\phi +\mu \right) & \frac{\beta \eta_{C}}{R_{0}}+\omega & \frac{\beta \eta_{A}}{R_{0}}+\alpha \\ 0 & \phi & -(\omega +\mu ) & 0\\ 0 & \rho & 0 & -(\alpha +\mu +\alpha ) \end{array})x^{*} \end{array}$$ (27)

where $x^{*}=x-\hat{x}$.

Now we can find the eigenvalues of the matrix in Eq (27) and hence the angular frequency, provided there are imaginary roots. Using Eq (27) and putting the values of the parameters, we can also find the angular frequency (*θ*) numerically. Hence the quasi-period (${\hat{T}}_{0}$, say) is obtained as ${\hat{T}}_{0}=\frac{2\pi }{\theta }$, which is independent of *N*. Now noting the relation $\hat{E}\left(\tau_{Q}\right)\;\text{log}\;2={\hat{T}}_{0}$ and using Result 1, we can solve for *N*.

Since we are dealing with a system consisting of four equations, it is not possible to obtain an explicit or compact expression for CCS (i.e. *N*) in terms of the model parameters. Hence we find an approximate value of CCS numerically.

Our approach is not restricted to only two [6] or three variables [5]. It generalizes the numerical calculation of CCS for any finite number of variables. Thus, our method for finding CCS is a general one and is applicable to any number of variables. Let *n* be the number of variables and *m* be the number of equations in the system of differential equations that explains the disease dynamics. An algorithm to calculate CCS for such a process is given below.

- **Step I**: Calculate basic reproduction number and endemic equilibrium for the system of differential equations i.e. the model.
- **Step II**: Write down a fully stochastic model and the joint distribution of the state variables conditioning on non-extinction of the disease.
- **Step III**: Write the expression for expected time to extinction based on quasi-stationary distribution.
- **Step IV**: Introduce diffusion approximation to obtain the local drift matrix and local covariance matrix from multivariate Ornstein-Uhlenbeck process.
- **Step V**: Using conditional truncated multivariate normal distribution, calculate expected time to extinction ($\hat{E}\left(\tau_{Q}\right)$, say)
- **Step VI**: Using linearisation of the model, calculate the angular frequency (${\hat{T}}_{0}$, say).
- **Step VII**: Merge expected time to extinction with angular frequency by the relation $\hat{E}\left(\tau_{Q}\right)\;\text{log}\;2={\hat{T}}_{0}$, and solve for *N*, which is the CCS with respect to the model.

Note that CCS thus calculated is an approximate one as we have made some approximations at few stages. However, this algorithm is a general one and may be applied to any model involving a system of differential equations that explains the disease dynamics. However, the basic reproduction number (*R*_0_) must be greater than 1 in order to calculate CCS using our method.

## Results

### Case study

For HIV transmission, we can calculate the critical community size for any region or community. Note that this value of CCS and time to extinction are approximate as we have made some approximation while applying diffusion approximation using conditional truncated multivariate normal distribution. All calculations are based on the assumed model (1)-(4). We suppose that an individual in the susceptible compartment/class (S) either moves to the infected (I) compartment or infected under treatment (C) compartment or infected with AIDS (A) compartment; while an individual from infected (I) class may receive treatment and move to infected under treatment (C) class or get infected with AIDS and move to infected with AIDS (A) class; while an individual after treatment may have reduced viral load and thus move from class C to the infected (I) class; similarly, an individual infected with AIDS (A) may have reduced viral load and move to infected (I) class. Death may occur in each class while immigration occurs only in the susceptible class.

It is clear that in order to calculate CCS for a region or community, we need to know the values of the parameters in the model. These parameter values are usually estimated based on different studies. As an illustration, we consider the values of the parameters based on the data available for Madagascar. However, the values of all parameters are not available in the literature. In such cases, we have assumed a few values considering the meaning and range of the parameters. Based on the available data for 2018 from the UNAIDS (www.unaids.org/en/regionscountries/countries/madagascar), we see that the number of people living with HIV who are on ART is 3500 whereas the number of adults and children living with HIV is 39000. The number of adults and children newly infected with HIV is 6100 and the number of deaths due to AIDS is 1700. Based on this information and following Nåsell [6] we have considered *β* = 0.156 × 365 = 56.94 and *d* = 0.044 × 365 = 16.06. Based on the available reports (http://cfs.hivci.org/country-factsheet.html) from the World Health Organisation (WHO), we have *ω* = 0.03 × 365 = 10.95, *ϕ* = 0.0035 × 365 = 1.2775, *η*_*C*_ = 0.000105, and *μ* = 0.015. Other parameters such as *α* = 0.001 × 365 = 0.365, *η*_*A*_ = 1.1, and *ρ* = 0.98 × 365 = 357.7 are based on available literature and intuitive meaning of the parameters. Given the poor socio-economic conditions of Madagascar, the 90-90-90 HIV treatment target initiative by UNAIDS has not performed remarkably well. At severe stages of HIV infection, available medication has very little effect. This information has led us to opt for such a high value of *ρ* and small value for *α*. Here, *β* is the contact rate (per year) of an HIV-infected person with the susceptible pool of individuals and *ρ* signifies the rate of progress (per year) of HIV-infected individuals to become AIDS patient. Similarly, we define other mentioned rates.

For this set of parameters, the CCS is obtained as 4585. So we can say that if the population in a commune drops below 4585, the infection will die out automatically. Obviously, the range of CCS for the HIV epidemic is far less than comparatively more infectious diseases such as measles that has much higher incidence rate. UNICEF reports that there were 244,607 cases of measles and 1,080 deaths in Madagascar from August 2018 to November 2019 (www.unicef.org/press-releases/measles-outbreaks-continue-unabated-five-countries-accounted-nearly-half-all-measles). Naturally, Nåsell [6] obtained CCS as 561, 000 (with *R*_0_ = 14) and Bartlett [4] estimated CCS as 250, 000 to 300, 000 in England and Wales for measles. Note that, using the above parameters for Madagascar and assuming homogenous mixing within communities, we obtain *R*_0_ as 4.059, which essentially means that in a completely susceptible population, a single infection may produce 4.059 secondary infections in the duration of infectiveness. Our calculation also indicates that temporary eradication or fade-out could happen after 7.365 years unless the infection is reintroduced from outside. This fade-out tends to happen in local spatial regions until the reintroduction of the disease occurs from other areas. However, this does not imply that a person already infected with HIV will be removed from the population within this period. In fact, she/he may continue to exist for more than 7.365 years and we assume that no new infection will be spread by her/him.

**Fig 1:**
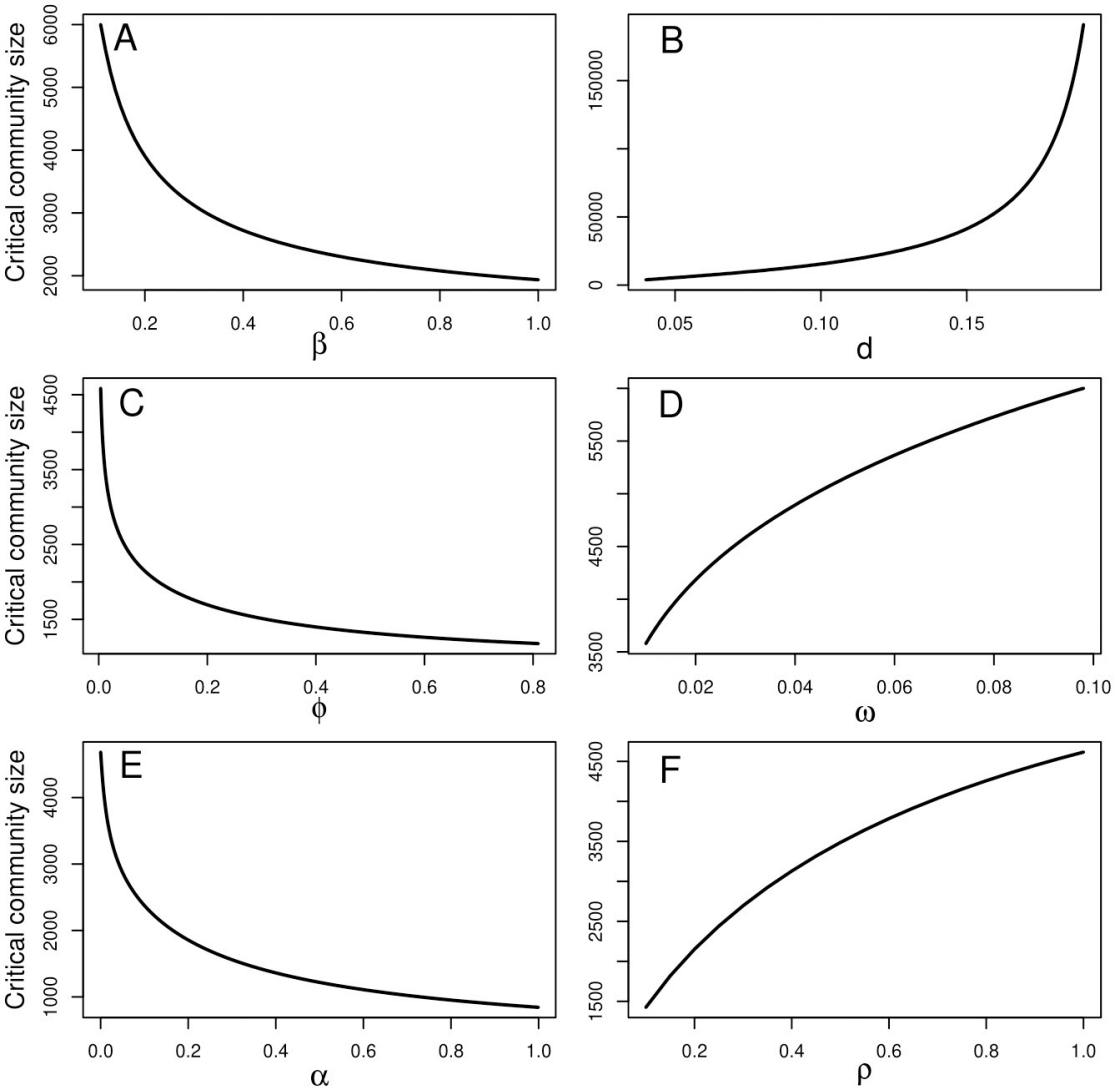
Relation of CCS with different parameters.

Next, we study the nature of CCS by varying one parameter and keeping others fixed at the above values. For this we consider the most important parameters (*β*, *d*, *ϕ*, *ω*, *α*, and *ρ*) in our model. Fig 1 indicates the relation of CCS with different parameters. Here, *β* is the contact rate for HIV transmission. An increase of *β* signifies an increment of infection circulation in the population. So the susceptible population will get infected at a much higher rate. In order to stop the infection, the population size should be very small. So if the number of the susceptible population falls below some threshold, the chance of the spread of infection will diminish. This indicates that smaller the population size, lesser would be the chance of spreading the infection. Thus, it is expected that if *β* increases, CCS will decrease (Fig 1A). *d* is the rate of death from class A. So as *d* increases, the spread of infection will decrease. For large values of *d*, a large susceptible population will remain unaffected. Hence the CCS value will be high for large *d* (Fig 1B). *ϕ* and *ω* respectively are the rates at which individuals in class *I* move to *C* and individuals in class *C* move to *I*. So, as *ϕ* increases, the number of individuals receiving treatment increases. It is expected that individuals under treatment will spread less infection than those who are infected but not treated. So, as more individuals go to class *I* from class *C*, the chance of spreading infection in the susceptible population decreases. But, it is known that the infection stays in a community long enough to engulf the entire susceptible population. Therefore, the infection might be eradicated if the susceptible population decreases. This would mean that, under the condition that *ϕ* increases, eradication of infection is expected to happen as the CCS value decreases (Fig 1C). On the contrary, as *ω* increases, CCS also increases (Fig 1D). Two other parameters viz. *α* and *ρ* represent the rates at which individuals in class *A* move to *I* and individuals in class *I* move to *A* respectively. For similar reasons, as *α* increases, CCS decreases (Fig 1E) but as *ρ* increases, CCS increases (Fig 1F).

## Discussion

Bartlett [3, 4] describes fade-out tendency of measles epidemic for small populations. He noticed that for such populations as the size of the susceptible pool of individuals is equal to CCS, measles has a 50% chance of fading out. Bartlett observed that for some cities in the United States where the population size is below CCS, the epidemic ceases to exist in absence of reintroduction of the infection from outside. But for larger cities, the tendency of damping down of successive epidemics is offset by random variability. The idea of epidemic fade-out in smaller spatial locations provides a clue to systematically treat epidemic rather than concentrating treatment for an entire continent or a large community at once.

Shocking statistics (www.prb.org/thestatusofthehivaidsepidemicinsubsaharanafrica) reveal that 16 countries in Sub-Saharan Africa constitute nearly 4% of the world’s population but account for more than 50% of HIV infections worldwide. In some of these countries, the prevalence rate is up to 30%. However, efforts to control HIV epidemic situations have resulted in a decrease in the disease prevalence in some African countries. Total eradication of highly prevalent diseases such as HIV infection in the context of countries in Sub-Saharan Africa is almost impossible in a very short time period. So we may instill the idea of CCS to systematically eradicate HIV. If we concentrate on the treatment of HIV in a more focussed manner in smaller spatial regions, it may accelerate the HIV annihilation process unless the disease is reintroduced from outside.

When we look at the geographical locations of Uganda, Malawi, Eswatini, and Madagascar, interestingly we find that being at the heart of the Great Lakes region, Uganda is surrounded by three lakes among which one is a fresh water lake. These lakes, to some extent separate Uganda from the rest of the African countries. Malawi again is separated by a freshwater lake (Lake Malawi) that comprises of 25% of its area. But Eswatini is a landlocked country in Southern Africa. We observe that, even with quite impressive progress towards achieving the 90-90-90 target, countries that are more landlocked have higher rates of incidence and prevalence compared to other countries that are isolated to varying extent. Although being in the AIDS belt of Africa, Madagascar has shown remarkably lower rates of incidence and prevalence even with little success of the 90-90-90 target. It is possibly because of the isolated location of Madagascar that separates it from the mainland of the African continent to a great extent.

Metcalf et al. [8] pointed out with respect to some childhood infections that the relative risk of extinction of infections on islands is almost double that in the mainland. Such infections cease to exist at a significantly higher rate in islands where access to/from the mainland is much less. But the persistence of infection within a region in the mainland is fuelled constantly by recolonization. Hence, although the epidemic infection should apparently die out because of the natural stochastic extinction property (damping down) of itself after invading the total susceptible subpopulation in the mainland, recolonization introduces infection and hinders the damping down process. Thus, our observations are consistent with the views of this paper.

Madagascar has 111 large and small cities or districts with 39.6% of them have population size less than 20000, 30.6% with population size somewhere between 20000 − 30000, 16.2% has 30000 − 40000 and 5.4% have population more than 100000 (http://worldpopulationreview.com/countries/madagascar-population/cities/). Further, each district is comprised of several communes of an even smaller population. So our idea is that if the population size falls below the CCS of Madagascar which is 4585 in each spatially separated population, HIV infection may cease to exist in different spatial locations, after a mean time of 7.365 years, provided infection is not re-introduced from outside. But this will definitely not happen without continuing the controlling measures like educating people about the disease and taking necessary preventive measures/treatment available. Through this paper, we are emphasizing that the systemic spreading of control measures could lead to an acceleration in achieving the 90-90-90 goal of UNAIDS.

We observe from our case study that in addition to the existing controlling measures of HIV epidemics in Madagascar, employing the idea of CCS might accelerate the eradication of the infection from smaller populations after the corresponding mean extinction time. Besides, CCS could be looked upon as an index of performance of epidemic controlling strategies in different countries.

## Conclusions

Combating pandemic and epidemic outbreaks require efficient controlling strategies, as the available treatment facilities and/or vaccines become limited in such situations. But, the infection persists in the community long enough to engulf the entire susceptible population. Hence, in large communities, the infection recurs in the subsequent timeline. Interestingly, Bartlett observed that in smaller communities the infection dies out if the susceptible population drops below a threshold size (CCS). Later, Nåsell formulated CCS for infectious diseases having Susceptible-Infected model dynamics. But the analytical calculation of CCS for more complex disease dynamics or higher-order models are quite cumbersome. So, we propose a simplified computation based approach for rapid calculation of the approximate value of CCS, for HIV disease dynamics with four differential equations. Since our method is free from any restriction on the number of variables in the system, it could be applied to other diseases with higher-order model dynamics. We envisage CCS for devising control strategies during epidemic outbreaks such as COVID-19 [18]. Because, during epidemic outbreaks with limited treatment facilities, keeping the susceptible population below CCS by enforcing appropriate containment plans might control the disease substantially.

So to sum up, through this paper, firstly, we have generalized the approach for CCS calculation for a system of differential equations having more than two variables. Our method for a system containing four variables may be extended to higher dimensions for any infectious disease dynamics. This is because our method is able to tackle multivariate normal distribution for calculating expected time to extinction as a function of CCS (Result 1). Secondly, we have developed a method to unleash the potential that CCS holds in devising control strategies by promptly determining its approximate numerical value from any disease dynamics using a simplified computation approach. In particular, we believe that our method will provide a suitable measure for accelerated extermination of epidemic infections by aiding policymakers in making decisions on control strategies. Thirdly, we hypothesize that, if we start treating spatially isolated regions or smaller communes and then progress to the district level, and after that to larger regions systematically, the annihilation of HIV infection could be accelerated. Although it is way difficult to reach out to smaller communes with treatments and other controlling measures (such as educating them about the infection etc.), CCS could be worth giving a trial, as this could utilize the natural force of damping down of epidemic oscillations to eradicate HIV infection from smaller populations.

## Supporting information

S1 Appendix (PDF)
