# Supplementary material for: Determination of critical community size from an HIV/AIDS model
Source: PLoS One. 2021 Jan 28;16(1):e0244543. doi: 10.1371/journal.pone.0244543 (PMC7842972; doi:10.1371/journal.pone.0244543)
Supplement: S1 Appendix — (PDF) [file pone.0244543.s001.pdf]

# Determination of critical community size from an HIV/AIDS model

Sarmistha Das[1], Pramit Ghosh[2], Sandip Banerjee[3], Saumyadipta Pyne[4,5], Joydev Chattopadhyay[6], Indranil Mukhopadhyay[1,*]

**1** Human Genetics Unit, Indian Statistical Institute, Kolkata, West Bengal, India
**2** Deben Mahata Government Medical College & Hospital, Purulia, West Bengal, India
**3** Department of Mathematics, Indian Institute of Technology, Roorkee, Uttarakhand, India
**4** Public Health Dynamics Lab and Department of Biostatistics, Graduate School of Public Health, University of Pittsburgh, Pittsburgh, Pennsylvania, USA
**5** Health Analytics Network, Pittsburgh, USA
**6** Agricultural and Ecological Research Unit, Indian Statistical Institute, Kolkata, West Bengal, India

* Corresponding author email: indranil@isical.ac.in

# Appendix

**Theorem 1** *Let* $\boldsymbol{Y} \sim N_p(\boldsymbol{\mu}, \Sigma)$ *and write* $\boldsymbol{y} = (\boldsymbol{y}_1', \boldsymbol{y}_2')'$, $\boldsymbol{\mu} = (\boldsymbol{\mu}_1', \boldsymbol{\mu}_2')'$, *and* $\Sigma = \begin{pmatrix} \Sigma_{11} & \Sigma_{12} \\ \Sigma_{21} & \Sigma_{22} \end{pmatrix}$ *Suppose instead of* $\mathbb{R}^p$, $\boldsymbol{Y}$ *is defined only on a truncated support* $\boldsymbol{c} < \boldsymbol{y} < \boldsymbol{d}$*. Consider the partitions* $\boldsymbol{c} = (\boldsymbol{c}_1', \boldsymbol{c}_2')'$ *and* $\boldsymbol{d} = (\boldsymbol{d}_1', \boldsymbol{d}_2')'$*. Then, the conditional distribution of* $\boldsymbol{Y}_1$ *given* $\boldsymbol{y}_2$ *is given by*

$$f^*(\boldsymbol{y}_1|\boldsymbol{y}_2) = \frac{f(\boldsymbol{y}_1|\boldsymbol{y}_2)}{\int_{\boldsymbol{c}_1}^{\boldsymbol{d}_1} f(\boldsymbol{y}_1|\boldsymbol{y}_2) d\boldsymbol{y}_1}$$

*where* $f(\boldsymbol{y}_1|\boldsymbol{y}_2)$ *is the conditional probability density function of* $\boldsymbol{Y}_1$ *given* $\boldsymbol{y}_2$ *i.e.* $\boldsymbol{Y}_1|\boldsymbol{y}_2 \sim N_q(\boldsymbol{\mu}_1 + \Sigma_{12}\Sigma_{22}^{-1}(\boldsymbol{y}_2 - \boldsymbol{\mu}_2), \Sigma_{11.2} = \Sigma_{11} - \Sigma_{12}\Sigma_{22}^{-1}\Sigma_{21})$

**Proof:** The joint p.d.f. of $\boldsymbol{Y}$ over the support $(\boldsymbol{c}, \boldsymbol{d})$ is given by,

$$\psi(\boldsymbol{y}) = k.f(\boldsymbol{y}) = kf(\boldsymbol{y}_1, \boldsymbol{y}_2)$$

where $k$ is such that,

$$k \int_{\boldsymbol{c}}^{\boldsymbol{d}} f(\boldsymbol{y})\, d\boldsymbol{y} = k \int_{\boldsymbol{c}_1}^{\boldsymbol{d}_1} \int_{\boldsymbol{c}_2}^{\boldsymbol{d}_2} f(\boldsymbol{y}_1, \boldsymbol{y}_2)\, d\boldsymbol{y}_1\, d\boldsymbol{y}_2 = 1$$

$$\implies k = \frac{1}{\int_{\boldsymbol{c}_1}^{\boldsymbol{d}_1} \int_{\boldsymbol{c}_2}^{\boldsymbol{d}_2} f(\boldsymbol{y}_1, \boldsymbol{y}_2)\, d\boldsymbol{y}_1\, d\boldsymbol{y}_2} = \frac{1}{\int_{\boldsymbol{c}_1}^{\boldsymbol{d}_1} \int_{\boldsymbol{c}_2}^{\boldsymbol{d}_2} f(\boldsymbol{y}_2) f(\boldsymbol{y}_1|\boldsymbol{y}_2)\, d\boldsymbol{y}_1\, d\boldsymbol{y}_2}$$

Again the marginal p.d.f. of $\boldsymbol{Y}_2$ is given by,

$$g(\boldsymbol{y}_2) = \int_{\boldsymbol{c}_1}^{\boldsymbol{d}_1} \psi(\boldsymbol{y})\, d\boldsymbol{y}_1 = k \int_{\boldsymbol{c}_1}^{\boldsymbol{d}_1} f(\boldsymbol{y}_1, \boldsymbol{y}_2)\, d\boldsymbol{y}_1 = kf(\boldsymbol{y}_2) \int_{\boldsymbol{c}_1}^{\boldsymbol{d}_1} f(\boldsymbol{y}_1|\boldsymbol{y}_2)\, d\boldsymbol{y}_1$$

Hence the conditional p.d.f. of $\boldsymbol{Y}_1$ given $\boldsymbol{y}_2$ is,

$$f^*(\boldsymbol{y}_1|\boldsymbol{y}_2) = \frac{\psi(\boldsymbol{y}_1, \boldsymbol{y}_2)}{g(\boldsymbol{y}_2)} = \frac{kf(\boldsymbol{y}_2)f(\boldsymbol{y}_1|\boldsymbol{y}_2)}{kf(\boldsymbol{y}_2)\int_{\boldsymbol{c}_1}^{\boldsymbol{d}_1} f(\boldsymbol{y}_1|\boldsymbol{y}_2)\, d\boldsymbol{y}_1} = \frac{f(\boldsymbol{y}_1|\boldsymbol{y}_2)}{\int_{\boldsymbol{c}_1}^{\boldsymbol{d}_1} f(\boldsymbol{y}_1|\boldsymbol{y}_2)\, d\boldsymbol{y}_1}$$

Hence, the result is proved. In particular, if $\boldsymbol{Y} \sim N_p(\boldsymbol{\mu}, \Sigma)$, then we know that $\boldsymbol{Y}_2 \sim N_{p-q}(\boldsymbol{\mu}_2, \Sigma_{22})$ and $\boldsymbol{Y}_1|\boldsymbol{y}_2 \sim N_q(\boldsymbol{\mu}_{1.2}, \Sigma_{11.2})$ where $\boldsymbol{\mu}_{1.2} = \boldsymbol{\mu}_1 + \Sigma_{12}\Sigma_{22}^{-1}(\boldsymbol{y}_2 - \boldsymbol{\mu}_2)$ and $\Sigma_{11.2} = \Sigma_{11} - \Sigma_{12}\Sigma_{22}^{-1}\Sigma_{21}$. Using the above result, we get the conditional distribution of $\boldsymbol{Y}_1$ given $\boldsymbol{y}_2$ on the truncated support.

**Result 1** *Let* $\phi(x) = \frac{1}{\sqrt{2\pi}}e^{-x^2/2}$ *and* $\Phi(x) = \int_{-\infty}^{x} \phi(t)dt$ *for any* $x \in (-\infty, \infty)$*. Then, an approximate expression of* $q_\bullet^{(d,\mu)}$ *is given as,*

$$q_\bullet^{(d,\mu)} = \frac{\mu p_{\bullet 100} + \mu p_{\bullet 010} + (d+\mu) p_{\bullet 001}}{1 - p_{\bullet 000}}$$

$$\text{where } p_{\bullet 100} \approx \frac{1}{2N\sqrt{\sigma_{22}^*}} \frac{\phi(\frac{\hat{x}_2^*}{\sqrt{\sigma_{22}^*}})}{\Phi(\frac{\hat{x}_2^*}{\sqrt{\sigma_{22}^*}})} . \frac{1}{2N\sqrt{\sigma_{33}^*}} \frac{\phi(\frac{\hat{x}_3^*}{\sqrt{\sigma_{33}^*}})}{\Phi(\frac{\hat{x}_3^*}{\sqrt{\sigma_{33}^*}})} . \frac{1}{2N\sqrt{\sigma_{44}}} \frac{\phi(\frac{\hat{x}_4}{\sqrt{\sigma_{44}}})}{\Phi(\frac{\hat{x}_4}{\sqrt{\sigma_{44}}})}$$

$$p_{\bullet 010} \approx \frac{1}{2N\sqrt{\sigma_{33}^{**}}} \frac{\phi(\frac{\hat{x}_3^{**}}{\sqrt{\sigma_{33}^{**}}})}{\Phi(\frac{\hat{x}_3^{**}}{\sqrt{\sigma_{33}^{**}}})} . \frac{1}{2N\sqrt{\sigma_{22}^{**}}} \frac{\phi(\frac{\hat{x}_2^{**}}{\sqrt{\sigma_{22}^{**}}})}{\Phi(\frac{\hat{x}_2^{**}}{\sqrt{\sigma_{22}^{**}}})} . \frac{1}{2N\sqrt{\sigma_{44}}} \frac{\phi(\frac{\hat{x}_4}{\sqrt{\sigma_{44}}})}{\Phi(\frac{\hat{x}_4}{\sqrt{\sigma_{44}}})}$$

$$p_{\bullet 001} \approx \frac{1}{2N\sqrt{\sigma_{44}^{***}}} \frac{\phi(\frac{\hat{x}_4^{***}}{\sqrt{\sigma_{44}^{***}}})}{\Phi(\frac{\hat{x}_4^{***}}{\sqrt{\sigma_{44}^{***}}})} . \frac{1}{2N\sqrt{\sigma_{22}^{***}}} \frac{\phi(\frac{\hat{x}_2^{***}}{\sqrt{\sigma_{22}^{***}}})}{\Phi(\frac{\hat{x}_2^{***}}{\sqrt{\sigma_{22}^{***}}})} . \frac{1}{2N\sqrt{\sigma_{33}}} \frac{\phi(\frac{\hat{x}_3}{\sqrt{\sigma_{33}}})}{\Phi(\frac{\hat{x}_3}{\sqrt{\sigma_{33}}})}$$

*where* $\hat{x}_i^*, \sigma_{ii}^*$ *for* $i = 2, 3$*,* $\hat{x}_i^{**}, \sigma_{ii}^{**}$ *for* $i = 2, 3$*,* $\hat{x}_i^{***}, \sigma_{ii}^{***}$ *for* $i = 2, 4$ *are obtained from the truncated conditional distribution of multivariate normal distribution as given in (23).*

**Proof:** First note that for large $N$, $\sqrt{N}(\boldsymbol{x} - \hat{\boldsymbol{x}})$ approximately follows a four-variate multivariate normal distribution with mean zero and covariate matrix $\Sigma$, as obtained from Eq (23). We also know that for small $h$ ($h > 0$), $\Phi(y + h) - \Phi(y) \approx h.\phi(y)$. Moreover, we shall show that $p_{\bullet 000}$, $p_{\bullet 100}$, $p_{\bullet 010}$, and $p_{\bullet 001}$ contain product of $\frac{\phi(\nu)}{\Phi(\nu)}$ terms. Since $N$ is unknown, we cannot evaluate its values exactly. Thus we use another approximation to $\frac{\phi(\nu)}{\Phi(\nu)}$ based on a logistic function only to make the calculation relatively simple. Putting $\sigma(z) = \frac{1}{1+e^{-z}}$ and $\beta = \frac{16}{15}\frac{\pi}{\sqrt{3}}$, for large $\nu$ we approximate $\frac{\phi(\nu)}{\Phi(\nu)}$ as,

$$\begin{aligned}\frac{\phi(\nu)}{\Phi(\nu)} =& \frac{\phi(\nu)}{\int_{-\infty}^{\nu} \phi(x)dx} = \frac{\beta\phi(\nu)}{\int_{-\infty}^{\beta\nu} \phi(\frac{y}{\beta})dy} \approx \frac{\beta\phi(\nu)}{\sigma(\beta\nu)} = \beta\phi(\nu)(1 + e^{-\beta\nu}) \\ \approx& \beta\Big[\frac{1 + cos(\nu)}{2\pi}\Big](1 + e^{-\beta\nu}) \approx \beta\frac{1 + cos(\nu)}{2\pi}\end{aligned}$$

Now using all these, we are able to evaluate an approximate expression of different

terms in $q_{\bullet}^{(d,\mu)}$ at equilibrium points.

$$
\begin{aligned}
p_{\bullet 100} =& \sum_{s=0}^{\infty} P(S=s, I=1, C=0, A=0) \\
\approx& P(0.5 < Nx_2(t) \leq 1, 0 < Nx_3(t) < 0.5, 0 < Nx_4(t) < 0.5) \\
=& P(0.5 < Nx_2(t) \leq 1 | 0 < Nx_3(t) < 0.5, 0 < Nx_4(t) < 0.5) \\
& \times P(0 < Nx_3(t) < 0.5 | 0 < Nx_4(t) < 0.5) P(0 < Nx_4(t) < 0.5) \\
=& \frac{\Phi(\frac{\frac{1}{N}-\hat{x}_2^*}{\sqrt{\sigma_{22}^*}}) - \Phi(\frac{\frac{1}{2N}-\hat{x}_2^*}{\sqrt{\sigma_{22}^*}})}{1-\Phi(\frac{\frac{1}{2N}-\hat{x}_2^*}{\sqrt{\sigma_{22}^*}})} \cdot \frac{\Phi(\frac{\frac{1}{2N}-\hat{x}_3^*}{\sqrt{\sigma_{33}^*}}) - \Phi(\frac{0-\hat{x}_3^*}{\sqrt{\sigma_{33}^*}})}{1-\Phi(\frac{0-\hat{x}_3^*}{\sqrt{\sigma_{33}^*}})} \cdot \frac{\Phi(\frac{\frac{1}{2N}-\hat{x}_4}{\sqrt{\sigma_{44}}}) - \Phi(\frac{0-\hat{x}_4}{\sqrt{\sigma_{44}}})}{1-\Phi(\frac{0-\hat{x}_4}{\sqrt{\sigma_{44}}})} \\
\approx& \frac{1}{2N\sqrt{\sigma_{22}^*}} \frac{\phi(\frac{\frac{1}{2N}-\hat{x}_2^*}{\sqrt{\sigma_{22}^*}})}{1-\Phi(\frac{\frac{1}{2N}-\hat{x}_2^*}{\sqrt{\sigma_{22}^*}})} \cdot \frac{1}{N\sqrt{\sigma_{33}^*}} \frac{\phi(\frac{0-\hat{x}_2^*}{\sqrt{\sigma_{33}^*}})}{1-\Phi(\frac{0-\hat{x}_3^*}{\sqrt{\sigma_{33}^*}})} \cdot \frac{1}{N\sqrt{\sigma_{44}}} \frac{\phi(\frac{0-\hat{x}_4}{\sqrt{\sigma_{44}}})}{1-\Phi(\frac{0-\hat{x}_4^*}{\sqrt{\sigma_{44}}})} \\
\approx& \frac{1}{2N\sqrt{\sigma_{22}^*}} \frac{\phi(\frac{\hat{x}_2^*}{\sqrt{\sigma_{22}^*}})}{\Phi(\frac{\hat{x}_2^*}{\sqrt{\sigma_{22}^*}})} \cdot \frac{1}{2N\sqrt{\sigma_{33}^*}} \frac{\phi(\frac{\hat{x}_3^*}{\sqrt{\sigma_{33}^*}})}{\Phi(\frac{\hat{x}_3^*}{\sqrt{\sigma_{33}^*}})} \cdot \frac{1}{2N\sqrt{\sigma_{44}}} \frac{\phi(\frac{\hat{x}_4}{\sqrt{\sigma_{44}}})}{\Phi(\frac{\hat{x}_4}{\sqrt{\sigma_{44}}})} \\
\approx& \frac{\beta^3}{64\pi^3 N^3 \sqrt{\sigma_{22}^* \sigma_{33}^* \sigma_{44}}} (1+\cos(\frac{\hat{x}_2^*}{\sqrt{\sigma_{22}^*}})(1+\cos(\frac{\hat{x}_3^*}{\sqrt{\sigma_{33}^*}}))(1+\cos(\frac{\hat{x}_4}{\sqrt{\sigma_{44}}}))
\end{aligned}
$$

$$
\begin{aligned}
p_{\bullet 010} =& \sum_{s=0}^{\infty} P(S=s, I=0.C=1, A=0) \\
\approx& P(0.5 < Nx_3(t) \leq 1 | 0 < Nx_2(t) < 0.5, 0 < Nx_4(t) < 0.5) \\
& \times P(0 < Nx_2(t) < 0.5 | 0 < Nx_4(t) < 0.5) P(0 < Nx_4(t) < 0.5) \\
=& \frac{\Phi(\frac{\frac{1}{N}-\hat{x}_3^{**}}{\sqrt{\sigma_{33}^{**}}}) - \Phi(\frac{\frac{1}{2N}-\hat{x}_3^{**}}{\sqrt{\sigma_{33}^{**}}})}{1-\Phi(\frac{\frac{1}{2N}-\hat{x}_3^{**}}{\sqrt{\sigma_{33}^{**}}})} \cdot \frac{\Phi(\frac{\frac{1}{2N}-\hat{x}_3^{**}}{\sqrt{\sigma_{33}^{**}}}) - \Phi(\frac{0-\hat{x}_2^{**}}{\sqrt{\sigma_{22}^{**}}})}{1-\Phi(\frac{0-\hat{x}_2^{**}}{\sqrt{\sigma_{22}^{**}}})} \cdot \frac{\Phi(\frac{\frac{1}{2N}-\hat{x}_4}{\sqrt{\sigma_{44}}}) - \Phi(\frac{0-\hat{x}_4}{\sqrt{\sigma_{44}}})}{1-\Phi(\frac{0-\hat{x}_4}{\sqrt{\sigma_{44}}})} \\
\approx& \frac{1}{2N\sqrt{\sigma_{33}^{**}}} \frac{\phi(\frac{\frac{1}{2N}-\hat{x}_3^{**}}{\sqrt{\sigma_{33}^{**}}})}{1-\Phi(\frac{\frac{1}{2N}-\hat{x}_3^{**}}{\sqrt{\sigma_{33}^{**}}})} \cdot \frac{1}{2N\sqrt{\sigma_{22}^{**}}} \frac{\phi(\frac{0-\hat{x}_2^{**}}{\sqrt{\sigma_{22}^{**}}})}{1-\Phi(\frac{\frac{1}{2N}-\hat{x}_2^{**}}{\sqrt{\sigma_{22}^{**}}})} \cdot \frac{1}{2N\sqrt{\sigma_{44}}} \frac{\phi(\frac{0-\hat{x}_4}{\sqrt{\sigma_{44}}})}{1-\Phi(\frac{\frac{1}{2N}-\hat{x}_4}{\sqrt{\sigma_{44}}})} \\
\approx& \frac{1}{2N\sqrt{\sigma_{33}^{**}}} \frac{\phi(\frac{\hat{x}_3^{**}}{\sqrt{\sigma_3^{**}}})}{\Phi(\frac{\hat{x}_3^{**}}{\sqrt{\sigma_{33}^{**}}})} \cdot \frac{1}{2N\sqrt{\sigma_{22}^{**}}} \frac{\phi(\frac{\hat{x}_2^{**}}{\sqrt{\sigma_2^{**}}})}{\Phi(\frac{\hat{x}_2^{**}}{\sqrt{\sigma_{22}^{**}}})} \cdot \frac{1}{2N\sqrt{\sigma_{44}}} \frac{\phi(\frac{\hat{x}_4}{\sqrt{\sigma_{44}}})}{\Phi(\frac{\hat{x}_4}{\sqrt{\sigma_{44}}})} \\
\approx& \frac{\beta^3}{64\pi^3 N^3 \sqrt{\sigma_{22}^{**} \sigma_{33}^{**} \sigma_{44}}} (1+\cos(\frac{\hat{x}_2^{**}}{\sqrt{\sigma_{22}^{**}}})(1+\cos(\frac{\hat{x}_3^{**}}{\sqrt{\sigma_{33}^{**}}}))(1+\cos(\frac{\hat{x}_4}{\sqrt{\sigma_{44}}}))
\end{aligned}
$$

$$
\begin{aligned}
p_{\bullet 001} &= \sum_{s=0}^{\infty} P(S=s, I=0, C=0, A=1) \\
&\approx P(0.5 < Nx_4(t) \leq 1 | 0 < Nx_2(t) < 0.5, 0 < Nx_3(t) < 0.5) \\
&\quad \times P(0 < Nx_2(t) < 0.5 | 0 < Nx_3(t) < 0.5) P(0 < Nx_3(t) < 0.5) \\
&= \frac{\Phi(\frac{\frac{1}{N} - \hat{x}_4^{***}}{\sqrt{\sigma_{44}^{***}}}) - \Phi(\frac{\frac{1}{2N} - \hat{x}_4^{***}}{\sqrt{\sigma_{44}^{***}}})}{1 - \Phi(\frac{\frac{1}{2N} - \hat{x}_4^{***}}{\sqrt{\sigma_{44}^{***}}})} \cdot \frac{\Phi(\frac{\frac{1}{2N} - \hat{x}_2^{***}}{\sqrt{\sigma_{22}^{***}}}) - \Phi(\frac{0 - \hat{x}_2^{***}}{\sqrt{\sigma_{22}^{***}}})}{1 - \Phi(\frac{0 - \hat{x}_2^{***}}{\sqrt{\sigma_{22}^{***}}})} \cdot \frac{\Phi(\frac{\frac{1}{2N} - \hat{x}_3}{\sqrt{\sigma_{33}}}) - \Phi(\frac{0 - \hat{x}_3}{\sqrt{\sigma_{33}}})}{1 - \Phi(\frac{0 - \hat{x}_3}{\sqrt{\sigma_{33}}})} \\
&\approx \frac{1}{2N\sqrt{\sigma_{44}^{***}}} \frac{\phi(\frac{\frac{1}{2N} - \hat{x}_4^{***}}{\sqrt{\sigma_{44}^{***}}})}{1 - \Phi(\frac{\frac{1}{2N} - \hat{x}_4^{***}}{\sqrt{\sigma_{44}^{***}}})} \cdot \frac{1}{2N\sqrt{\sigma_{22}^{***}}} \frac{\phi(\frac{0 - \hat{x}_2^{***}}{\sqrt{\sigma_{22}^{***}}})}{1 - \Phi(\frac{\frac{1}{2N} - \hat{x}_2^{***}}{\sqrt{\sigma_{22}^{***}}})} \cdot \frac{1}{2N\sqrt{\sigma_{33}}} \frac{\phi(\frac{0 - \hat{x}_3}{\sqrt{\sigma_{33}}})}{1 - \Phi(\frac{\frac{1}{2N} - \hat{x}_3}{\sqrt{\sigma_{33}}})} \\
&\approx \frac{1}{2N\sqrt{\sigma_{44}^{***}}} \frac{\phi(\frac{\hat{x}_4^{***}}{\sqrt{\sigma_{44}^{***}}})}{\Phi(\frac{\hat{x}_4^{***}}{\sqrt{\sigma_{44}^{***}}})} \cdot \frac{1}{2N\sqrt{\sigma_{22}^{***}}} \frac{\phi(\frac{\hat{x}_2^{***}}{\sqrt{\sigma_{22}^{***}}})}{\Phi(\frac{\hat{x}_2^{***}}{\sqrt{\sigma_{22}^{***}}})} \cdot \frac{1}{2N\sqrt{\sigma_{33}}} \frac{\phi(\frac{\hat{x}_3}{\sqrt{\sigma_{33}}})}{\Phi(\frac{\hat{x}_3}{\sqrt{\sigma_{33}}})} \\
&\approx \frac{\beta^3}{64\pi^3 N^3 \sqrt{\sigma_{22}^{***}\sigma_{33}\sigma_{44}^{***}}} (1 + \cos(\frac{\hat{x}_2^{***}}{\sqrt{\sigma_{22}^{***}}}) (1 + \cos(\frac{\hat{x}_3}{\sqrt{\sigma_{33}}})) (1 + \cos(\frac{\hat{x}_4^{***}}{\sqrt{\sigma_{44}^{***}}}))
\end{aligned}
$$

$$
\begin{aligned}
p_{\bullet 000} &= \sum_{s=0}^{\infty} P(S=s, I=0, C=0, A=0) \\
&\approx P(0 < Nx_2(t) < 0.5 | 0 < Nx_3(t) < 0.5, 0 < Nx_4(t) < 0.5) \\
&\quad \times P(0 < Nx_3(t) < 0.5 | 0 < Nx_4(t) < 0.5) P(0 < Nx_4(t) < 0.5) \\
&= \frac{\Phi(\frac{\frac{1}{N} - \hat{x}_2^{*}}{\sqrt{\sigma_{22}^{*}}}) - \Phi(\frac{\frac{1}{2N} - \hat{x}_2^{*}}{\sqrt{\sigma_{22}^{*}}})}{1 - \Phi(\frac{\frac{1}{2N} - \hat{x}_2^{*}}{\sqrt{\sigma_{22}^{*}}})} \cdot \frac{\Phi(\frac{\frac{1}{N} - \hat{x}_3^{*}}{\sqrt{\sigma_{33}^{*}}}) - \Phi(\frac{\frac{1}{2N} - \hat{x}_3^{*}}{\sqrt{\sigma_{33}^{*}}})}{1 - \Phi(\frac{\frac{1}{2N} - \hat{x}_3^{*}}{\sqrt{\sigma_{33}^{*}}})} \cdot \frac{\Phi(\frac{\frac{1}{N} - \hat{x}_4}{\sqrt{\sigma_{44}}}) - \Phi(\frac{\frac{1}{2N} - \hat{x}_4}{\sqrt{\sigma_{44}}})}{1 - \Phi(\frac{\frac{1}{2N} - \hat{x}_4}{\sqrt{\sigma_{44}}})} \\
&\approx \frac{1}{2N\sqrt{\sigma_{22}^{*}}} \frac{\phi(\frac{\frac{1}{2N} - \hat{x}_2^{*}}{\sqrt{\sigma_{22}^{*}}})}{1 - \Phi(\frac{\frac{1}{2N} - \hat{x}_2^{*}}{\sqrt{\sigma_{22}^{*}}})} \cdot \frac{1}{2N\sqrt{\sigma_{33}^{*}}} \frac{\phi(\frac{0 - \hat{x}_3^{*}}{\sqrt{\sigma_{33}^{*}}})}{1 - \Phi(\frac{\frac{1}{2N} - \hat{x}_3^{*}}{\sqrt{\sigma_{33}^{*}}})} \cdot \frac{1}{2N\sqrt{\sigma_{44}}} \frac{\phi(\frac{0 - \hat{x}_4}{\sqrt{\sigma_{44}}})}{1 - \Phi(\frac{\frac{1}{2N} - \hat{x}_4}{\sqrt{\sigma_{44}}})} \\
&\approx \frac{1}{2N\sqrt{\sigma_{22}^{*}}} \frac{\phi(\frac{\hat{x}_2^{*}}{\sqrt{\sigma_{22}^{*}}})}{\Phi(\frac{\hat{x}_2^{*}}{\sqrt{\sigma_{22}^{*}}})} \cdot \frac{1}{2N\sqrt{\sigma_{33}^{*}}} \frac{\phi(\frac{\hat{x}_3^{*}}{\sqrt{\sigma_{33}^{*}}})}{\Phi(\frac{\hat{x}_3^{*}}{\sqrt{\sigma_{33}^{*}}})} \cdot \frac{1}{2N\sqrt{\sigma_{44}}} \frac{\phi(\frac{\hat{x}_4}{\sqrt{\sigma_{44}}})}{\Phi(\frac{\hat{x}_4}{\sqrt{\sigma_{44}}})} \\
&\approx \frac{\beta^3}{64\pi^3 N^3 \sqrt{\sigma_{22}^{*}\sigma_{33}^{*}\sigma_{44}}} (1 + \cos(\frac{\hat{x}_2^{*}}{\sqrt{\sigma_{22}^{*}}}) (1 + \cos(\frac{\hat{x}_3^{*}}{\sqrt{\sigma_{33}^{*}}})) (1 + \cos(\frac{\hat{x}_4}{\sqrt{\sigma_{44}}}))
\end{aligned}
$$
